# Effect of process variables on the production of Polyhydroxyalkanoates by activated sludge

**DOI:** 10.1186/1735-2746-9-6

**Published:** 2012-09-07

**Authors:** Nader Mokhtarani, Hossein Ganjidoust, Ebrahim Vasheghani Farahani

**Affiliations:** 1 Civil and Environmental Engineering Faculty, Tarbiat Modares University, Tehran, Iran; 2 Chemical Engineering Faculty, Tarbiat Modares University, Tehran, Iran

**Keywords:** Acetate, Activated sludge, Biodegradable plastics, Polyhydroxyalkanoates, Sludge retention time, Taguchi, Volatile fatty acids

## Abstract

Polyhydroxyalkanoates are known to be temporarily stored by microorganisms in activated sludge, especially in anaerobic-aerobic processes. Due to the problems resulted from the disposals of plastic wastes and excess sludge of wastewater treatment plants, the production of polyhydroxyalkanoates by treating activated sludge and determining the effect of process variables were the main issues of this paper. In this research, an anaerobic-aerobic sequencing batch reactor was used to make microorganism adapted and a batch aerobic reactor was used for enriching them. The variables affecting polyhydroxyalkanoates production including aeration time, sludge retention time, and volatile fatty acids concentration of the influent in sequencing batch reactor, and also carbon to nitrogen ratio and cultivation time in polymer production reactor, were investigated using Taguchi statistical approach to determine optimum conditions. The maximum polymer production of 29% was achieved at sludge retention time of 5–10 days, aeration time of 2 hours, supplementation of 40% of volatile fatty acids in the influent and increasing of carbon to nitrogen ratio of polymer production reactor to above 25 g/g. Based on the results, in optimum conditions, the volatile fatty acids concentration which increased the production of polyhydroxyalkanoates up to 49% was the most effective variable. Carbon to nitrogen ratio, sludge retention time and aeration time were ranked as the next affecting parameters. Although the polyhydroxyalkanoates content achieved in present study is much lower than that by pure culture, but the proposed method may still serve well as an environmental friendly means to convert waste into valuable product.

## Introduction

Polymers are known to play an important role in our life because of their desirable properties. Current concerns about the environmental fate of polymeric materials have created much interest in the development of biodegradable polymers.

Among the various biodegradable plastics, polyhydroxyalkanoates (PHAs) are attractive substitutes for conventional petrochemical plastics. PHAs, which are intracellular storage materials, have many potential applications in medicine, agriculture, pharmacy, and packaging due to their biodegradability and biocompatibility. They accumulate as distinct inclusions in the cell and comprise up to 80% of the dry cell weight for strains of *ralstonia eutropha* (previously known as *alcaligenes eutrophus*), under conditions of nitrogen or phosphate limitation and excess carbon source [1,2]. Production and recovery of poly-Β-hydroxyl-butyrate from whey degradation by Azotobacter was examined by Khanafari et al. [3]. In this research after 48 h, in presence of meat extract as the source of nitrogen, poly-β hydroxyl-butyrate production was increased up to 75% of the cell dry weight.

Polymers produced from PHAs have been reported to be completely biodegradable [4,5]. In addition, because they are produced from renewable resources, they are attractive to human beings as environmentally friendly materials [6]. Even though PHAs have been recognized as good candidates for biodegradable polymers, their high price compared with that of petrochemical-based polymers has limited their use in a wide range of applications [7,8].

The production of biodegradable plastic on a large scale is limited because of the relative expense of the substrate, low polymer production, and the cost of extraction and recovery. According to Yamane [9], higher costs, especially raw material cost, make it difficult for PHAs biodegradable plastics to compete with conventional petroleum-based plastics in the commercial market place. Almost 30% of total PHAs production cost is attributed to the carbon source [10]. The use of inexpensive feed-stocks for competitive bioplastics (PHAs) production was recommended by Gonzalez-Lopez et al. [11]. Ramos-Cormenzana et al. [12] used olive-mill wastewater (alpechin) for biodegradable plastics production.

A good candidate for economical PHAs production would be a mixed culture (such as activated sludge) that can store high PHAs content while growing on an inexpensive substrate. Nowadays a great amount of excess sludge is generated daily in the world. Additionally, about 40 to 60 percent of the investment expenses of the activated sludge treatment plants have to do with treating the sludge coming from the wastewater treatment plants [13-15]. One possible strategy for excess sludge management is reutilization of sludge as useful resources.

During last two decades, many researchers have been investigating PHA_S_ production by using activated sludge. Mino et al. [16] used the activated sludge acclimatized in the anaerobic-aerobic process for enhanced biological phosphate removal to accumulate PHA_S_. Satoh et al. [17] studied PHA_S_ production by using activated sludge and compared it with that produced by pure culture. Chua et al. [18] studied the effect of acetate concentration in influent, pH and sludge retention time (SRT) on the production of PHA_S_ by activated sludge treating municipal wastewater. Fermentative volatile fatty acids (VFAs) as carbon sources to synthesize PHA_S_ by activated sludge were examined by Cai et al. [19]. In another study, it was found that activated sludge from a pulp and paper industry wastewater treatment plant had the best potential for PHA_S_ production among activated sludge from municipal, industrial starch, and industrial dairy wastewater treatment plants, in laboratory scale experiments [20]. Release of intracellular polymeric substances from activated sludge caused the increment of sludge dewatering capability and was considered as an effective method for sludge volume reduction [21].

**Figure 1:**
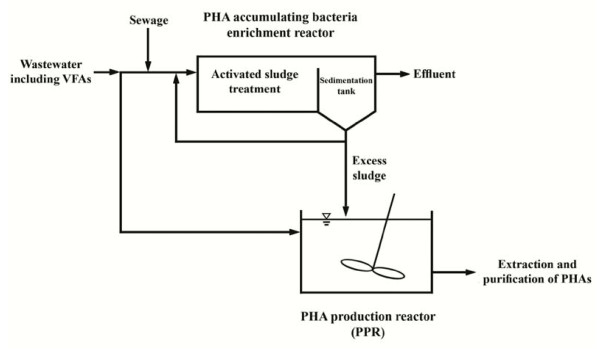
**The proposed PHA**_**S**_** production system by activated sludge.**

The production system of PHAs as biodegradable plastics by activated sludge that inspired from Takabatake et al. [22] is shown in Figures 1. In this system at first, activated sludge with high potential of PHAs production was acclimatized in “PHAs accumulating bacteria enrichment reactor”. In this stage, it was essential to optimize the operational conditions for sludge acclimatization or for the enrichment of PHAs accumulating microorganisms, so that the PHAs production capability of activated sludge could be improved. In the second step, the acclimatized activated sludge was transferred into PHA production reactor (PPR). In this reactor, wastewater with high concentration of VFAs was fed as carbon sources for PHAs production. 

In the present work, our focus was mainly on the optimization of operational conditions and determining the effect of each process variable. For this purpose, an anaerobic-aerobic sequencing batch reactor (SBR) and a fully aerobic batch process were selected for microorganism acclimatization and PHAs production, respectively. The operational conditions being investigated were the carbon to nitrogen ratio (C: N) of influent, cultivation time in PPR, aeration time, SRT, and VFAs concentration of influent in SBR.

## Materials and methods

### Operation of SBR under different operational condition

**Figure 2:**
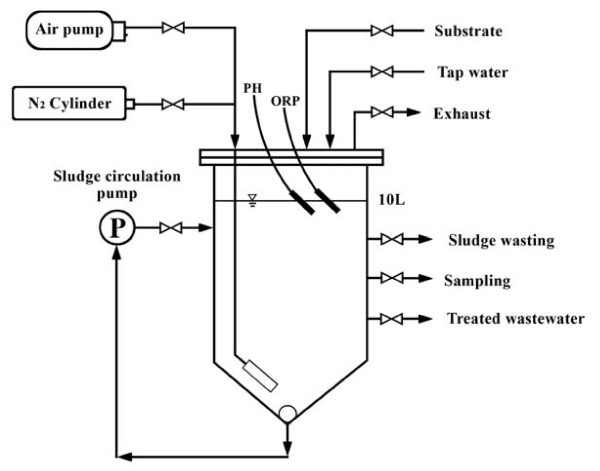
Schematic diagram of SBR.

The schematic of a 10-liter SBR, which was operated in the anaerobic-aerobic mode, is shown in Figures 2. It was operated with synthetic wastewater with the specification shown in Table 1 as the influent.

**Table 1:** Characterization of wastewater employed

**Parameter**	**Unit**	**Range**
MgCl_2_	mg/L	60
NH_4_Cl	mg/L	49
CaCl_2_	mg/L	14
KCl	mg/L	59
K_2_HPO_4_	mg/L	44
Acetic acid	mg/L	98
Sodium propionate	mg/L	109
Urea	mg/L	13
Sucrose	mg/L	112
Peptone	mg/L	84
Yeast extract	mg/L	31

In order to change VFA_S_ concentration in the substrate solution, carbon sources were replaced with the desired amount of sodium acetate and sucrose. The reactor was put in a room with constant temperature of 23-25°C. The origin of the seed was sludge of municipal wastewater treatment plant in Tehran.

The SBR was operated continuously in cycles of 4, 5, or 6 hours. One standard cycle consisted of 15 min for decantation, 5 min for influent feeding, 10 min for nitrogen purging, 1 hour for anaerobic, 2, 3 or 4 hours for aerobic and 30 min for sedimentation periods. Substrate solution was added at the end of nitrogen purging phase. In the aerobic phase, air was supplied by an aeration pump to obtain the minimum dissolved oxygen concentration of 2 mg/ L.

The SRT was controlled at three different operational conditions of 5, 10, and 15 days. For each change of SRT at least three times of the selected SRT was considered for adaptation. A sludge circulation pump in the anaerobic phase and the supply of air under the aerobic conditions provided the mixing of activated sludge mixed liquor. The SBR was completely automated and controlled by an electrical control box.

### PHA_S_ production reactor

**Figure 3:**
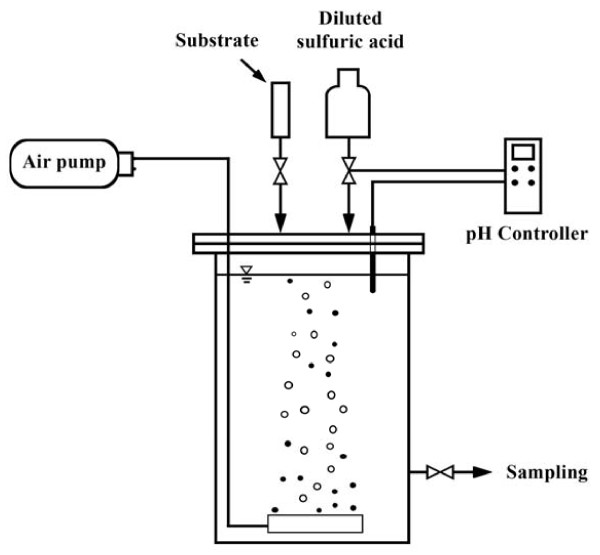
Schematic diagram of PPR.

In order to measure the PHA_S_ production potential of activated sludge, batch experiments were conducted under aerobic conditions. Activated sludge was taken at the end of aerobic phase of SBR, and put in a glass jar as shown in Figures 3. Sodium acetate was added as carbon source for PHA_S_ production and incubated in aerobic condition. Based on preliminary test for acetate uptake rate (not shown in this paper), in each experiment 500 mg/L carbon source was added at t = 0, and then 250 mg /L at t = 6, 12 and 18 hours. In this reactor, pH had a tendency to increase and was kept between 7–7.5 by adding diluted sulfuric acid.

To change carbon to nitrogen ratio in the influent of PPR, ammonium sulfate was used as the nitrogen source. K_2_HPO_4_ was also added in order to supply phosphate requirement of the system. The batch experiments were conducted under the same temperature as SBR.

In all batch experiments, one sample was taken at 0, 6, 12, 18 and 24 hours, respectively, for analysis. Addition of acetate and sampling from PPR during different periods of time were also completely automated.

### Analytical methods

All parameters except PHAs were analyzed according to APHA, AWWA, WEF [23]. PHAs concentration of suspension (activated sludge mixed liquor) was determined by gas chromatography (GC) after methanolytic decomposition of activated sludge in acidified hot chloroform [24]. The GC used for this purpose (Philips-PU4410) was equipped with a capillary column (BP-5, SEG. Austria, 25 m length, 220 μm internal diameter, less than 1 μm film thickness) and a flame ionization detector. The detector and injector temperature were adjusted to 200°C. Initial temperature setting of column was 100°C for 2 min., then increased in 8°C/min to 140°C and was maintained for 5 min. One micro liter of sample was split injected into the GC column (split ratio was 1:25). Sodium 3-hydroxybutirate (Sigma, USA) and copolymer (88:12 wt%) of 3-hydroxybutirate (3HB) and 3-hydroxyvalerate (3HV) (Sigma, USA) were used as the standard for the quantification of 3HB and 3HV, respectively. As acetate was used as the carbon source in this study, the monomeric unit of the produced PHAs was almost all 3HB. All other chemicals employed for analysis were of analytical grade and obtained from reliable companies.

### Design of experiment and analysis of variance

Production of PHA_S_ by activated sludge process is affected by numerous variables. In this research, to investigate the effect of different factors (including: aeration time, SRT, VFAs content of influent and C: N of PPR) and to determine the optimum conditions of the process, the statistical Taguchi approach was applied [25].

Based on the results of other researchers, 3 levels were selected for each of four chosen factors [18,26]. The considered levels for these variables in an L_9_ orthogonal array are given in Table 2. Each experiment (trial) was repeated tree times, and the average of corresponding results in terms of the percentage of PHA_S_ production is also shown in this Table. 

**Table 2:** **Orthogonal L**_**9**_** array and results of experiments**

**Factor**					
**Trial**	**Aeration time (h)**	**SRT(day)**	**VFA**_**S**_**(%)**	**C:N(g/g)**	**PHA**_**S**_**(%)**
1	2	5	0	10	9
2	2	10	40	25	29
3	2	15	100	N = 0	21
4	3	5	40	N = 0	25
5	3	10	100	10	17
6	3	15	0	25	10
7	4	5	100	25	21
8	4	10	0	N = 0	12
9	4	15	40	10	10

The analysis of the experimental results by this method is expressed in terms of the main effects of each factor, which is defined as the average of the results for a factor at a specified level.

## Results

### Effect of cultivation time

**Figure 4:**
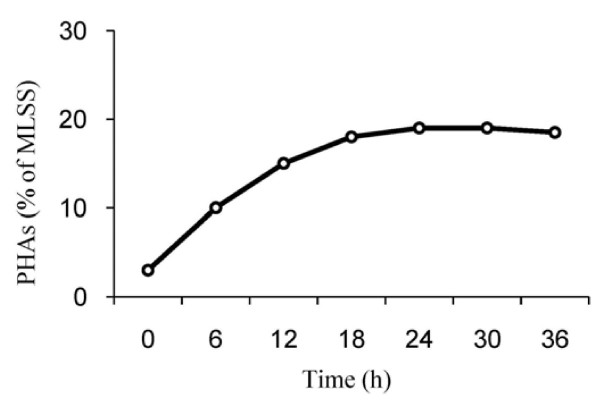
Effect of cultivation time on the percentage of PHAs produced.

To study the effect of cultivation time of activated sludge on the PHA_S_ production potential, preliminary experiments were carried out at 23-25°C for periods of 0–30 hours. The relationship between cultivation time and PHA_S_ production, which was positive, is shown in Figures 4. As shown, accumulation of PHA_S_ into the cell walls increased with increasing of cultivation time. Based on these result, although maximum productivity happened after 18 hours, but a cultivation time of 24 hours was chosen for further experiments.

### Analysis of the results by the Taguchi method

**Figure 5:**
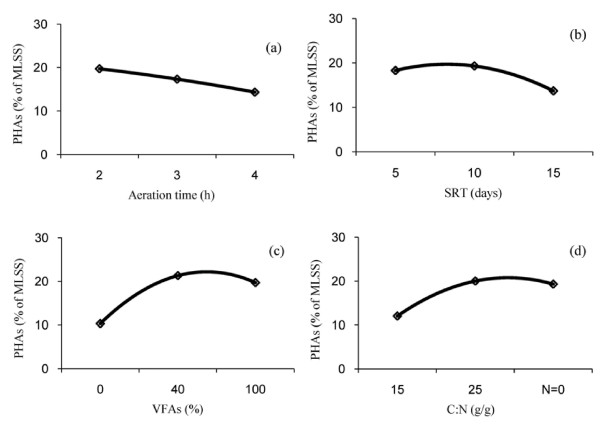
Main effects of variables on the PHAs production: (a) Aeration time; (b) SRT; (c) VFAs; (d) C:N.

To study the effect of process variables on the PHA_S_ production by activated sludge, the statistical Taguchi method was applied for the design of the experiments as described before. The percentages of PHA_S_ production, given in Table 2 for each experiment, were used to calculate the main effects of the factors. These results are shown in Figures 5 and will be discussed in the following section. The analysis of the results is also summarized in Table 3. According to the analysis of variance, in optimum conditions, VFA_S_ which increased the production of PHAs up to 49% was the most effective variable. C:N of PPR influent, sludge retention time and aeration time with about 28%, 13% and 10% increasing of polymer production, were ranked as the next responsibles.

**Table 3:** Analysis of variance for PHAs production (%) in Taguchi design

**Factor**	**DOF**	**Sum of squares**	**Variance**	**Percent**
Aeration time (h)	2	42.888	21.444	10.046
SRT (day)	2	54.889	27.444	12.857
VFAs (%)	2	210.888	105.444	49.401
C:N (g/g)	2	118.222	59.111	27.693
Error	0			
Total				100

## Discussion

As shown in Figures 5a, the PHAs production capability of activated sludge acclimatized in SRT of 5 days decreased with increasing of aeration time from 2 to 4 hours. Same trend was observed for both SRTs of 10 and 15 days (Figure not shown).

The reason why at longer aeration time less PHAs was produced, is that as aeration time increases the substrate concentration decreases and microorganisms are forced to declining growth and then endogenous phase [27]. In this phase not only the microbial population is reduced but also microbial communities, which are dominants in the system, may change. Therefore, longer aeration time may select microbial community with lower PHAs production capacity than that selected under shorter aeration times. At lower than 2 hours aeration time, the system may be at log-growth phase. During this period an excess amount of food exists and the population of microorganisms is less than that in stationary phase. As a result, activated sludge processes operating in stationary phase can produce more PHAs compared to that with endogenous or log-growth phases. Based on these results, the optimum aeration time for PHAs production for this system was chosen to be 2 hours.

Figures 5b shows that increasing of SRT up-to 5 days had considerable effect on the PHAs production. However, a further increase of SRT had no significant effect on polymer production ability. The initial increase of PHAs production capability can be related to the difference in organic loading to biomass. Dionisi et al. [28] showed in their anoxic batch tests that a maximum PHAs storage might occur at intermediate organic loading rate. A practically stable trend attained for PHAs production by further increase of sludge retention time can be possibly attributed to the reduction of sludge yield under a longer SRT [18]. On the basis of these results, although there was no significant difference between PHAs production predicted (by Software) in SRTs of 5 to 10 days, but 10 days was selected as the optimum SRT for PHAs production.

The effect of VFAs content of SBR feed on PHAs production is shown in Figures 5c. It seems that supplementation of 40% acetate in influent wastewater considerably improved the PHAs production capability of activated sludge, but a further increase of VFAs had not considerable effect on the PHAs production. These results suggest that, in acetate-rich acclimatization, the PHAs storage capacity of microorganisms might be increased, thus leading to a higher PHAs production capacity. It is therefore suggested that the wastewater containing volatile fatty acids should be used to enhance the PHAs production capability of acclimatized sludge.

As shown in Figures 5d increasing of C: N up to 25 g/g increased PHAs production, but further increase of C:N on the PHAs production was not considerable. This behavior can be reasoned as follows:

1-Coexistence of nitrogenous compounds increases acetate uptake rate of activated sludge. However, it is used to promote the productivity of structural materials such as intracellular carbohydrates (ICH) and protein [22].

2-Further increasing of nutrient concentration in the substrate may act as the limiting material for cell synthesis and growth [27], as well as for acetate uptake rate and PHAs production.

On the basis of these results, 25 g/g was selected as optimum C:N for the PHAs production.

Based on the results of this study, the optimum conditions for the production of PHAs using activated sludge were 2 hours for aeration time, 5 to 10 days of SRT, supplementation of 40% of VFAs in the influent of SBR, and more than 25 g/g of C:N for PPR. The optimum condition coincides with trial (experiment No.2 in Table 2), at which the PHAs production was 29%. Although the PHAs content achieved in present study was much lower than that by pure culture, but the proposed method may still serve well as an environmental friendly means to convert waste into valuable product.

## Competing interests

The authors declare that they have no competing interests.

## Authors’ contributions

The overall implementation of this study including design, experiments and data analysis, and manuscript preparation were the results of efforts by Corresponding author. All authors have made extensive contribution into the review and finalization of this manuscript. All authors read and approved the final manuscript.
